# Rac1 activation links tau hyperphosphorylation and Aβ dysmetabolism in Alzheimer’s disease

**DOI:** 10.1186/s40478-018-0567-4

**Published:** 2018-07-13

**Authors:** Mirta Borin, Claudia Saraceno, Marcella Catania, Erika Lorenzetto, Valeria Pontelli, Anna Paterlini, Silvia Fostinelli, Anna Avesani, Giuseppe Di Fede, Gianluigi Zanusso, Luisa Benussi, Giuliano Binetti, Simone Zorzan, Roberta Ghidoni, Mario Buffelli, Silvia Bolognin

**Affiliations:** 1 Department of Neurosciences, Biomedicine and Movement Sciences, University of Verona, Strada Le Grazie, 8, 37134 Verona, Italy; 2 Molecular Markers Laboratory, IRCCS Istituto Centro San Giovanni di Dio Fatebenefratelli, Via Pilastroni, 4, 25125 Brescia, Italy; 3 Division of Neurology 5 and Neuropathology, IRCCS Foundation - Carlo Besta Neurological Institute, Via Celoria 11, 20133 Milan, Italy; 4 MAC Memory Center, IRCCS Istituto Centro San Giovanni di Dio Fatebenefratelli, Brescia, Italy; 5 Environmental Research and Innovation (ERIN) Department, Luxembourg Institute of Science and Technology (LIST), Belvaux, L-4422 Luxembourg; 6 Luxembourg Centre for Systems Biomedicine, University of Luxembourg, 6 avenue du Swing, L-4367 Belvaux, Luxembourg

## Abstract

One of the earliest pathological features characterizing Alzheimer’s disease (AD) is the loss of dendritic spines. Among the many factors potentially mediating this loss of neuronal connectivity, the contribution of Rho-GTPases is of particular interest. This family of proteins has been known for years as a key regulator of actin cytoskeleton remodeling. More recent insights have indicated how its complex signaling might be triggered also in pathological conditions. Here, we showed that the Rho-GTPase family member Rac1 levels decreased in the frontal cortex of AD patients compared to non-demented controls. Also, Rac1 increased in plasma samples of AD patients with Mini-Mental State Examination < 18 compared to age-matched non demented controls. The use of different constitutively active peptides allowed us to investigate in vitro Rac1 specific signaling. Its activation increased the processing of amyloid precursor protein and induced the translocation of SET from the nucleus to the cytoplasm, resulting in tau hyperphosphorylation at residue pT181. Notably, Rac1 was abnormally activated in the hippocampus of 6-week-old 3xTg-AD mice. However, the total protein levels decreased at 7-months. A rescue strategy based on the intranasal administration of Rac1 active peptide at 6.5 months prevented dendritic spine loss. This data suggests the intriguing possibility of a dual role of Rac1 according to the different stages of the pathology. In an initial stage, Rac1 deregulation might represent a triggering co-factor due to the direct effect on Aβ and tau. However, at a later stage of the pathology, it might represent a potential therapeutic target due to the beneficial effect on spine dynamics.

**Electronic supplementary material:**

The online version of this article (10.1186/s40478-018-0567-4) contains supplementary material, which is available to authorized users.

## Introduction

Alzheimer’s disease (AD) is an age-associated disorder, characterized by the abnormal depositions of hyperphosphorylated tau protein in the form of neurofibrillary tangles (NFT) and of β-amyloid (Aβ) peptide in the form of senile plaques (SP). These thoroughly studied hallmarks are certainly key contributors to the development of the pathology. However, it is the synaptic and dendritic loss, which seems to be the best predictor of the clinical symptoms. This dysfunction has been extensively described in early studies [45, 57, 64] and confirmed in more recent years [17, 42, 59]. Reduction in spine number correlates well with the degree of memory loss and cognitive impairment of the patients [16].

Despite the plethora of evidence showing that the loss of spine stability is associated with AD, the identification of pathways responsible for this abnormal disruption is still elusive. In physiological conditions, spine morpho-dynamics rely on changes of F-actin-rich cytoskeleton, which are highly regulated by Rho-GTPases (see reviews [10, 27]). Rho-GTPases maintain the equilibrium between actin monomer (G-actin) and filament (F-actin) pools [18]. This family of proteins comprises of three main members: Ras-related C3 botulinum toxin substrate 1 (Rac1), cell division protein 42 (Cdc42), and Ras homologous member A (RhoA). By switching between an active GTP and inactive GDP-bound form, they convert signals of the postsynaptic receptors into changes of actin binding proteins, ultimately resulting in the remodeling of spine shape and density [15, 47]. The signaling pathway is quite complex: the timely activation of the 3 proteins is strictly regulated by the association to several additional regulatory proteins, which are in turn activated during synaptic transmission or neurotrophic factor release [47, 58, 65].

As impaired actin cytoskeleton stability [6, 48], including the formation of actin rod-like inclusions [33], has been shown in AD brains, Rho-GTPase signaling deregulation might contribute to the synaptic degeneration observed in the disease [1, 10]. Among the different members, Rac1 has shown to be connected to amyloid precursor protein (APP) processing. Studies on hippocampal primary neurons showed that Rac1-specific inhibitor decreased APP protein levels in a concentration-dependent manner by modulating its transcriptional activity [67]. However, studies examining the direct connection between Aβ and Rac1 are contradictory, leaving a rather unclear scenario regarding the potential contribution of the protein to disease-relevant mechanisms [34, 44, 52].

More puzzling is the connection with the other key pathological hallmark of the pathology, tau hyperphosphorylation. In the context of cancer and cell migration studies, Rac1 was shown to directly bind the oncoprotein SET [61, 63]. Interestingly for the AD field, SET is the inhibitor of the protein phosphatase 2A (PP2A), the major regulator of tau phosphorylation. The laboratory of Dr. Khalid Iqbal showed extensively how SET abnormally translocated from the nucleus to the cytoplasm in the brain of AD patients compared to controls [62]. In the cytosol, SET can directly bind PP2A and decrease its activity [4]. The pathological relevance of this pathway was demonstrated by the fact that the overexpression of SET by adeno-associated viral vectors generated a rat model of sporadic AD [9, 68, 69].

Here, we provide evidence showing that Rac1 was altered in fronto-cortical brain lysate and plasma of AD patients compared to healthy age-matched controls. Importantly, the degree of the alteration in the circulating Rac1 pool reflected the severity of the cognitive impairment, suggesting a potential role of Rac1 as a biomarker for AD. In vitro studies on mouse primary cortical neurons and SH-SY5Y showed that the triggering of selective Rac1 signaling induced the generation of pathogenic Aβ fragments and the translocation of SET from the nucleus to the cytoplasm. This resulted in an increase of tau phosphorylation (at pT181). Active Rac1 increased in 6-week-old 3xTgAD mouse hippocampus while the total level decreased at 7 months compared to controls. Intranasal treatment with a constitutively active form of the peptide at 6.5 months resulted in a rescue of the number of dendritic spines compared to vehicle-treated animals.

## Materials and methods

### Human subjects

Brain samples were provided by the Biobank of the IRCCS Foundation – Carlo Besta Neurological Institute and from the Brain Bank of the Department of Pathology at Indiana University School of Medicine. We included 24 brains from AD patients and 12 from age-matched non-demented controls (Table 1). The neuropathological diagnosis was performed according to international guidelines for the assessment of AD [25].

**Table 1:** Braak stage and known co-pathologies of the brain samples in the AD study group

ID	Braak stage	Co-pathology
AD 1	VI	Cerebrovascular disease
AD 2	VI	Hemorrage
AD 3	VI	–
AD 4	VI	Lewy body
AD 5	V	Cerebrovascular disease
AD 6	V-VI	–
AD 7	VI	–
AD 8	III-IV	–
AD 9	VI	–
AD 10	VI	Lewy body
AD 11	VI	–
AD 12	III-IV	Lewy body
AD 13	IV	–
AD 14	III [31]	–
AD 15	V-VI	Hemorrhage
AD 16	VI	–
AD 17	VI	Vascular formation
AD 18	VI	Cerebrovascular disease
AD 19	VI	Cerebrovascular disease
AD 20	V-VI	Cerebrovascular disease
AD 21	V-VI	Cerebrovascular disease
AD 22	VI	Diffuse Lewy body
AD 23	V-VI	–
AD 24	IV	–

For the plasma samples, the patients considered for this study underwent clinical and neurological examination at the MAC Memory Center of the IRCCS Centro San Giovanni di Dio-Fatebenefratelli, Brescia. Clinical diagnosis of AD, MCI was made according to international guidelines [37, 38, 51]. We included AD (*n* = 114) and MCI (*n* = 47) patients, and age and sex-matched cognitively healthy controls (CTRL, *n* = 102). Biological samples were collected and stored in the Biobank of the IRCCS Centro San Giovanni di Dio-Fatebenefratelli, Brescia, Italy, after obtaining informed consent, as approved by the local ethics committee (approval No. 26/2014). The study was approved by the local ethics committee (approval No. 03/2015). Plasma was isolated according to standard procedures. The demographic characteristics of the patients in the study are shown in Tables 2, 3.

**Table 2:** Demographic characteristic and plasma Rac1 levels in the four groups

	Controls	MCI	AD MMSE≥18	AD MMSE< 18
No. of subject	102	47	72	42
Gender (% female)	49	64	60	48
Mean age (SD), years	70 (5)	75 (6)	72 (5)	73 (6)
Rac1 (ng/ml) range, median and mean	0.10–1.82 0.38, 0.45	0.16–8.21 0.42, 0.77	0.14–6.81 0.37, 0.62	0.16–6.95 0.76, 1.00
MMSE (SD)	28 (1)	26 (2)	22 (2)	9 (6)

**Table 3:** Demographic characteristic and plasma RhoA levels in the four groups

	Controls	MCI	AD MMSE≥18	AD MMSE< 18
No. of subjects	83	45	47	27
Gender (% female)	52	64	58	41
Mean age (SD), years	70 (5)	75 (6)	72 (5)	73 (5)
RhoA (pg/ml) range, median and mean	3.26–1634 27.82, 153.7	1.78–849 28.77, 98.17	1.38–812.3 56.35, 132.5	2.96–1395 38.8, 144.1
MMSE (SD)	28 (1)	26 (2)	22 (2)	8 (7)

### Cell culture and drug/peptide treatments

Primary neuronal cultures (from E18 C57BL/6 J mouse embryos) and SH-SY5Y neuroblastoma cells were obtained and cultured as previously described [11]. For the okadaic acid (OA) treatment, the powder (Sigma-Aldrich) was dissolved in dimethylsulfoxide (DMSO, Sigma-Aldrich) at 50 μM concentration as stock solution. In working solutions, DMSO never exceeded the concentration of 0.02% and the final OA concentration was 10 nM. The TAT fusion proteins were prepared as previously described [12]. For a scheme of the recombinant mutant proteins with all the mutations and a complete amino acid sequence, see Lorenzetto et al. [30]

For the Leptomycin B (LMB, #9676 Cell Signaling Technology) treatment, SH-SY5Y cells were differentiated to a mature neuron-like phenotype by retinoic acid (RA, R2625 Sigma-Aldrich). Cells were seeded on a glass slide (12 mm Ø), at a density of 10^3^ cells per well, and RA was added 3 h after plating at a final concentration of 10 μM in DMEM with 2% FBS and maintained for 10 days. LMB was added to the medium at a final concentration of 10 pM for 48 h, alone or together with Tat-Rac1L61F37A or Tat-Rac1WT (1 μM).

The MTT assays were performed 3 or 4 times, in triplicate as previously described [11]. The various treatments were compared to the control (untreated cells), which represented 100% viability.

### Animal housing and intranasal delivery

Animal breeding and handling were performed following a protocol approved by the Animal Care and Use Committee of the University of Verona (CIRSAL), and authorized by the Italian Ministry of Health, in strict adherence to the European Communities Council directives (86/609/EEC). Mice were housed with water and food ad libitum and with 12 h/12 h light/dark cycle, under standard environmental conditions (temperature, humidity). For these studies, female 3xTg-AD mice harboring APP_swe_, PS1_M146V_, tau_P301L_ transgenes [43], and age-matched control (C57BL/6) were purchased from the Jackson laboratory (New Harbor, ME, USA), and used at 4 different ages: 6 week-, 3, 7, and 16 month-old.

Intranasal administration is a non-invasive method for delivering therapeutic agents to the central nervous system. Animals were randomly assigned to the two treatment groups (PBS solution, Veh; Rac1-L61F37A mutant peptide solution (100 μM)). Mice were anesthetized with isofluorane, and a total volume of 16 μl solution was administered (alternating smaller injections of 4 μl each to the left and to the right nares with 10 min between each administration). Animals were treated 3 times a week for two weeks, starting from 6.5 months.

### ELISA analyses

Brain samples from frontal cortex of AD patients and age-matched non-demented controls were homogenized in 9 volumes of 1X PBS using a manual Dounce homogenizer and centrifuged at 1500 xg for 15 min. Supernatants were collected and the total protein amount was measured by BCA Protein Assay kit (Pierce).

Blood samples from AD and MCI patients and CTRL were kept at 4 °C for at least 20 min and then centrifuged for 5 min (4 °C, 1,000×g). Plasma was collected and centrifuged 5 min (4 °C, 1,000×g) after the addition of the protease inhibitors.

Rac1 and RhoA levels were measured in plasma, in duplicate, using commercially available ELISA kits (Human Ras-Related C3 Botulinum Toxin Substrate 1, RAC1, and Human Transforming Protein RhoA, MyBioSource).

Rac1-GTP level was assayed using Rac1 Activation Biochem Kit™ (#BK035, Cytoskeleton). Proteins were separated by SDS-PAGE and, after blotting, membranes were probed with anti-Rac1 antibody (mouse anti-Rac1, 1:1000, #05–389, Upstate). GAPDH antibody (rabbit anti-GAPDH, 1:20000, #G9545, Sigma-Aldrich) was used as a loading control. All the kits were used according to the manufacturer’s instructions.

### Immunoprecipitation and immunoproteomic analysis

The mouse brain dissection was performed in a plastic petri dish on ice, after collecting the whole brain from the mouse skull. The two cortexes and the hippocampus were collected, flash frozen in liquid nitrogen, and stored at − 80 °C until analysis. The whole procedure did not exceed 5 min to preserve brain integrity.

Brain homogenates (10% weight/volume) were obtained using a micro-pestle on ice in cold lysis buffer containing: 50 mM Tris-HCl (pH 7.5), 2% Igepal, 10 mM MgCl_2_, 0.5 M NaCl, 2 mM ethylenediaminetetraacetic acid (EDTA), 2 mM ethylene glycol tetraacetic acid (EGTA), 5 mM benzamidine, 0.5 mM phenylmethylsulfonyl fluoride (PMSF), 8 mg/mL pepstatin A and 20 mg/mL leupeptin, 50 mM b-glycerolphosphate, 100 mM sodium fluoride, 1 mM sodium vanadate, 20 mM sodium pyrophosphate, and 100 nM OA. Homogenates were clarified by a centrifugation at 4 °C (10000xG 1 min). After assessment of protein concentration by Precision red protein quantification assay (Cytoskeleton #ADV02), lysates were processed for either Rac1 activation assay (Cytoskeleton # BK035) or Western Blot. For the Rac1 activation assay, lysates were diluted to a concentration of 0.5 mg/ml.

The following primary antibodies were used: GluR1 (Anti-GluR, Recombinant rabbit monoclonal antibody, 1:250, 05-855R, Millipore/Merk); Lamin B1 (mouse monoclonal antibody, 1:1000, B-10:sc-374,015, Santa Cruz Biotechnology); PSD95 (anti-PSD 95 mouse monoclonal antibody, 1:1000, #124 01, Synaptic Systems); pT181 (Phospho-Tau (Thr181) mouse monoclonal antibody (AT270), 1:1000, MN1050 Thermo Fisher Scienific); Tau-5 (Mouse (monoclonal) Anti-tau (Neurofibrillary Tangles Marker), clone Tau-5, 1:1000, Invitrogen/Thermo Fisher Scienific, USA); TuJ1 (rabbit anti-ß-tubulin III antibody, 1:2500, T2200 Sigma-Aldrich).

Primary cortical neurons were seeded into 6-well plates, at a density of 9,5 × 10^5^ cells per well. Following treatments, cells were washed 1× in Tris-buffered saline (TBS), then lysed and scraped with 50 μl of pre-warmed Laemmli buffer and boiled for 10 min. The cell lysate was assayed for protein using the Bradford method (Sigma-Aldrich). The lysates were separated using a 4–12% Bis-Tris gels (Novex pre-cast gel, Invitrogen) and transferred to 0.45 μm nitrocellulose membrane (Invitrogen) for probing with antibodies. Blots were blocked for 1 h at room temperature in 1X Odyssey blocking buffer (TBS) and incubated with primary antibodies overnight in Odyssey blocking buffer (TBS) plus 0.1% Tween-20 at 4 °C. Then the membranes were washed 3 × 10 min in TBST (Tween-20 TBS) at room temperature, followed by incubation with secondary antibody conjugated to IRDye diluted in Odyssey blocking buffer (TBS) plus 0.1% Tween-20 for 1 h at room temperature. Blots were washed 2 × 10 min in TBST, 1 × 10 min in TBS and visualized with Odyssey Infrared Imaging System.

Levels of total tau and tau phosphorylation at each specific site were determined by using phosphorylation-dependent and site-specific tau antibodies from Invitrogen (rabbit anti-tau (pS262) phosphospecific antibody, 1:1000 for WB and 1:100 IF, #44-750G; mouse anti-tau, 1:1000, #AHB0042; rabbit anti-tau (pS202) phosphospecific antibody 1:1000 for WB and 1:100 IF, #44779G). The pan-Actin antibody (mouse anti-actin, 1:2500, #MAB1501, Millipore or rabbit anti-actin, 1:2500, #A2066, Sigma-Aldrich) was used as a loading control. Primary antibodies were detected using anti-mouse IRDye 800 (1:2500; Li-Cor) or anti-rabbit Alexa Flour 680 (1:5000; Invitrogen). Blots were scanned and subsequently quantified using the Odyssey Imaging System (Li-Cor) by quantifying fluorescent signals as Integrated Intensities (I.I. K Counts) using the Odyssey Infrared Imaging System, Application Version 1.2 software. After background subtraction, ratios were calculated for each antibody against the pan-actin loading control using I.I. K Counts. The respective antibody to pan-actin ratio was then used to calculate phosphorylated protein to total protein ratio.

The subcellular fractionation on primary cortical neurons was performed as previously described [74]. For these experiments, 5-6 × 10^6^ neurons were seeded. The membrane fraction was not quantified but directly suspended in 15 μl of sample buffer and loaded on the gel.

### Surface-enhanced laser desorption/ionization time-of-flight mass spectrometry (SELDI TOF MS)

Immunoproteomic analyses of Aβ isoforms were performed as previously described with minor modifications [2]. Briefly, 3 μl of the specific monoclonal human antibodies (mAbs) 4G8 (anti-Aβ_17–24_) + 6E10 (anti-Aβ_1–16_) (BioLegend, San Diego, CA, USA) at the total mAbs concentration of 0.125 mg/ml (concentration of each mAbs was 0.0625 mg/ml), were incubated in a humidity chamber for 3 h at room temperature (RT) to allow covalent binding to the PS20 ProteinChip Array (Bio-Rad, Laboratories, Inc.). Unreacted sites were blocked with Tris-HCl 0.5 M, pH 8 in a humid chamber at RT for 1 h. Each spot was washed first 3 times with PBS containing 0.5% (*v*/v) Triton X-100 and then twice with PBS. The spots were coated with 5 μl of cell lysate and incubated in a humid chamber at 4 °C overnight. Each spot was washed first 3 times with PBS containing 0.1% (v/v) Triton X-100, twice with PBS, and finally with deionized water. One microliter of α-ciano-4-hydroxy cinnamic acid (CHCA, Bio-Rad Laboratories, Inc.) was added to each spot. Mass identification was made using the ProteinChip SELDI System, Enterprise Edition (Bio-Rad Laboratories, Inc.). The analysis was performed with mass focus FM5500 and laser energy E1500.

### Golgi staining and spine count

Animals were sacrificed by terminal anaesthesia with 2,2,2-Tribromoethanol (ip dose of 0,8 g/kg body weight; T48402, Sigma-Aldrich, Germany), and intracardially perfused with 0.9% saline solution added with 0.5% heparin (H3393, Sigma-Aldrich) to remove blood, followed by 4% paraformaldehyde buffered pH 7, to fix brain tissue. Brains were impregnated in 50 fold volume of staining solution, containing 1% HgCl_2_ (Sigma-Aldrich, n°7,487,947, Germany), 1% K_2_Cr_2_O_7_ (Sigma-Aldrich) and 1% K_2_CrO_4_ (Sigma-Aldrich), and stored at room temperature for 2 weeks in the dark.

Brain sections (80 to 100 μm thickness) were obtained using a vibratome (Leica VT1200, Leica Biosystems, Germany). Sections were placed in a mixture consisting of 1 part Developer Replenisher solution (GBX n° 3,101,508, Carestream Dental) and 2 parts Milli-Q water for 5 min, rinsed in Milli-Q water for 5 min, placed in a mixture consisting of 1 part Fixer Replenisher solution (GBX n° 3,101,557, Carestream Dental) and two parts Milli-Q water for 15 min, and then rinsed again in Milli-Q water for 5 min. Sections were then dehydrated in 60, 80 and 100% ethanol 2 min each, cleared in 100% xylene for 2 min and then mounted in Eukitt (03989 Sigma-Aldrich). Dendrites and spines of 4 sub regions of the cortex (primary motor cortex, secondary motor cortex, posterior parietal area and visual cortex) were imaged by a 100X oil objectives using Olympus BX63 microscope (Olympus Corporation, Japan) and acquired by Neurolucida 64-Bit software (MBF Bioscience, USA). Dendritic spines were manually counted scrolling along the z-stack (0.35 μm) of acquired images, and tagging spines using the multi-point selection tool of ImageJ 1.47v software (NIH, USA). The dendritic length was determined using the ImageJ software segmented line tool.

### Immunostaining and confocal analysis

Immunocytochemistry and image acquisition was performed as previously described [11]. The following primary antibodies were used: anti-active Rac1 (1:1000, #26903, NewEast), pan-axonal neurofilament marker (1:1000, #SMI-312R, Covance), rhodamine phalloidin (1:40, #R415, Thermo Fisher Scientific), Aβ_17–24_ (1:200, 4G8; #SIG-39200, Covance), anti-map2 (1:500, #M9942, Sigma-Aldrich), anti-SET (I2PP2A; 1:50, #sc-25,564, Santa Cruz Biotechnology), site-specific tau antibodies were purchased from Invitrogen (1:100, #44779G).

### Aβ_1–42_ oligomer preparation and dot-blot

The preparation of Aβ1_− 42_ synthetic oligomers was performed according to a previously described protocol [32]. The supernatant with Aβ_1–42_ oligomers was assayed for protein content using the Bradford kit (Sigma-Aldrich). The oligomerization of Aβ_1–42_ was checked by dot blotting using two different antibodies: 6E10 (beta amyloid antibody; #SIG-39320, Covance) and A11 (anti-oligomer antibody; #AHB0052, Invitrogen). 0.1 to 1 μg of each oligomeric preparation were applied on a nitrocellulose membrane and allowed to air dry. The membrane was then washed with TBS for 5 min and blocked with Odyssey Blocking Buffer (Li-Cor, #FE3092750000) for 1 h at room temperature. The membranes were then incubated overnight at 4 °C with 6E10 (1:2000) or the conformation dependent antibody A11 (1:500) in Odyssey Blocking Buffer with 0.1% Tween-20. Following 3 10-min washes, the blot was incubated with secondary antibody (anti-mouse IRDye 800, 1:2500 (Li-Cor) or anti-rabbit Alexa Flour 680, 1:5000 (Invitrogen)) for 1 h at room temperature, washed again and scanned on Odyssey Imaging System (Li-Cor).

### Regulatory context of Rac1 and AD by bioinformatics tools

The role of Rac1 in the AD was investigated starting from the genes related to the disease through GWAS (Genome Wide Association Studies). The GWAS Catalog [70] allowed collecting 720 genes statistically linked to the pathology. In order to reconstruct a network connecting the selected genes, including others likely involved in the process, we started from ANAT [3]. ANAT is a bioinformatics tool to chart molecular pathways including direct high confidence interactors to connect all the input genes. SET and PP2A were added to the list. Of the GWAS list, ANAT did not recognize 269 genes. The resulting network was enriched by ANAT with 182 high confidence interactors connecting GWAS nodes, including Rac1.

### Statistical analysis

Data were analysed with Prism 5 (GraphPad Software). Statistical significant differences are reported as *p < 0.05, **p < 0.01, and ***p < 0.001. The correlation of plasma Rac1 with MMSE was performed using the Spearman’s correlation procedure with SPSS 20.0 software for Windows (IBM). The sample size and the used statistical tests are indicated in Table 4.

**Table 4:** Sample size and performed statistical analysis

Figure Number	Experiment	Test	Sample size (n)	*P* value
Fig. 1a	Rac1 AD human brain	Mann Whitney	12, 24	0.028
Fig. 1b	Rac1 human AD plasma	Kruskal-Wallis Dunn’s test	102, 47, 72, 42	0.0005 CTRL vs AD MMSE< 18 p = 0.0002; MCI vs AD MMSE< 18 p = 0.045; AD MMSE≥18 vs AD MMSE< 18p = 0.0051;
Fig. 3b	SET/GluR1	Two-tailed One sample t test	10,10,10,6,6	CTRL vs Rac1-WT *p* = 0.039 CTRL vs Rac1-L61F37A, *p* = 0.037
Fig. 3d	pT181/GAPDH	Two-tailed One sample t test	9, 7, 7,3,3	CTRL vs Rac1-WT *p* = 0.023; CTRL vs Rac1-L61F37A, *p* = 0.014
Fig. 3d	pT181/Tau5	Two-tailed One sample t test	9, 7, 7,3,3	CTRL vs Rac1-L61F37A, p = 0.045;
Fig. 5b	Hippocampus 6 weeks Rac1GTP/Rac1	Student t test	4, 4	0.044
Fig. 5c	Cortex 7 months Rac1/GAPDH	Student t test	6, 6	0.005
Fig. 6a	PSD95/Tuj1	One-way Anova Turkey’s MC	8, 9, 8	0.0064 C57 + Veh vs 3xTgAD + Veh * 3xTgAD + Veh vs 3xTgAD + Rac1 *
Fig. 6d	Spine density	One-way Anova Turkey’s MC	4, 4, 4	0.0061 C57 + Veh vs 3xTgAD + Veh * 3xTgAD + Veh vs 3xTgAD + Rac1 **
Additional file 1: Figure S2A	Aβ toxicity	Two-tailed One sample t test	4, 4, 4	Aβ0.1 μM 0.0044 Aβ0.5 μM 0.0088 Aβ1μM 0.0414
Additional file 1: Figure S4C	3 h OA	Two-Tailed paired t test	6, 6	0.003
Additional file 1: Figure S4C	6 h OA	Two-Tailed paired t test	4, 4	0.038

*p < 0.05; **p < 0.01

## Results

### Rac1 protein levels are altered in human AD fronto-cortical brain and plasma samples

**Fig. 1:**
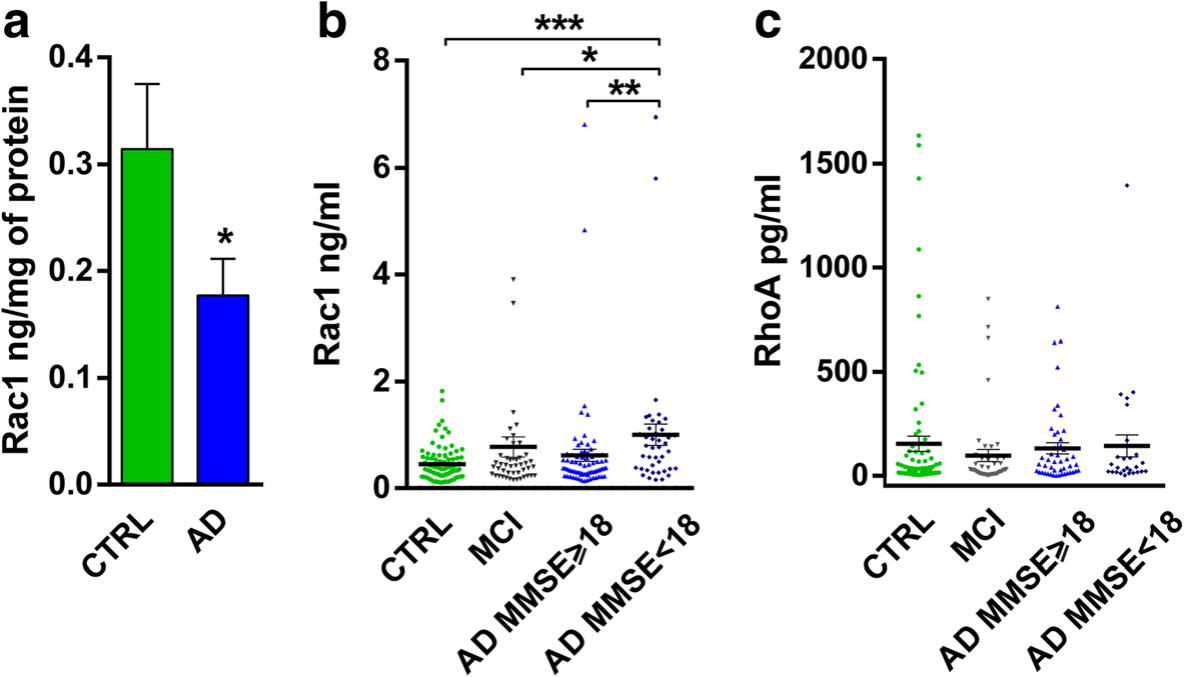
Rac1 is altered in AD brain and plasma samples. **a** Rac1 (ng/mg of protein) was measured in brain homogenates from CTRL subjects and AD patients. **b** Rac1 (ng/ml of protein) was measured in plasma samples from CTRL subjects, MCI, and AD patients (MMSE≥18 and MMSE< 18). **c** RhoA (pg/ml of protein) was measured in plasma samples from CTRL subjects, MCI, and AD patients. The data represented are mean ± SEM

To investigate the role of Rac1 in the pathogenesis of AD, fronto-cortical brain homogenates from 24 neuropathologically confirmed AD patients and 12 age-matched non-demented controls were analysed. Rac1 levels decreased in AD brains as compared to controls (Fig. 1a). We also evaluated Rac1 protein levels in the plasma of 114 patients affected by AD, 47 subjects with mild cognitive impairment (MCI), and 102 sex and age-matched non-demented controls. To investigate the link between Rac1 and cognitive decline, a correlation analysis was performed between Rac1 levels and the Mini-Mental State Examination (MMSE) in AD: Rac1 plasma levels were negatively correlated with MMSE score (*r* = − 0.208; *p* = 0.026). We stratified AD patients based on their MMSE score (AD patients with MMSE< 18, *n* = 42; AD patients with MMSE≥18, *n* = 72). Rac1 levels significantly increased in the plasma of the AD patients with MMSE< 18 compared to controls (*p* = 0.0002), MCI (*p* = 0.045), and the AD group with MMSE≥18 (*p* = 0.0051) (Kruskal-Wallis followed by Dunn’s multiple comparison test) (Fig. 1b). No alterations were detected in RhoA plasma levels in AD patients and MCI subjects (AD MMSE≥18 *n* = 47; AD MMSE< 18 *n* = 27; MCI *n* = 45; CTRL 83; *p* = 0.104 Kruskal-Wallis test) (Fig. 1c).

### Rac1 perturbation affects APP metabolism

To modulate Rac1 activity, we generated TAT-Rac1 mutant proteins. These mutants contained a sequence coding for TAT, derived from the 86-amino acid transactivation protein involved in HIV replication, which allows the internalization of the protein into the cell. The produced proteins were: (i) Rac1-WT, which contained the wild type sequence of the protein; (ii) Rac1-L61F37A and Rac1-L61Y40C, two double mutants with a point mutation, which tonically activated the protein (Q61L, abbreviated in L61), and a second point mutation (F37A or Y40C), which conferred selectivity to the downstream signalling delivery [28]; (iii) Rac1-N17, a dominant negative (DN) mutant with a single point mutation (T17 N, abbreviated as N17).

**Fig. 2:**
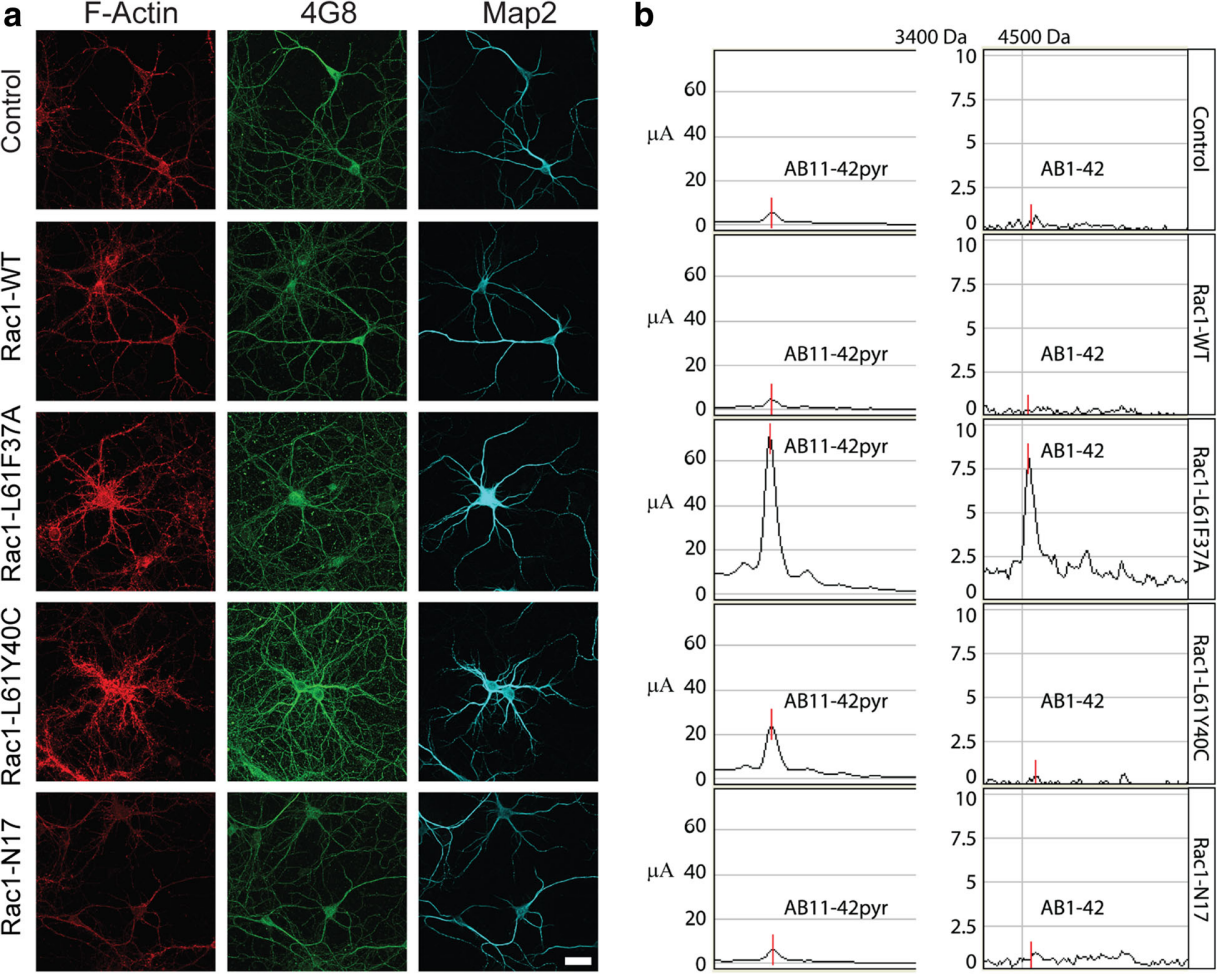
Rac1 mutant peptides interfere with APP metabolism. **a** Primary cortical neurons treated between DIV11 and DIV14 with Rac1 mutant peptides. After 24 h, cells were stained to visualize of F-actin, APP and Aβ (4G8), and dendrites (MAP2). The increase in F-actin fluorescence was considered as a sign of Rac1 activation. These representative images were obtained from one of four independent experiments. Scale bar 30 μm. **b** Representative spectra of Aβ isoforms from SH-SY5Y cell lysates treated with TAT-Rac1 mutant proteins or vehicle (*n* = 2 experiments in triplicate). Peak intensity is expressed in μA (μAmpere). 3400 and 4500 are m/z reference values expressed in Dalton in the spectrum scale (m/z for Aβ 11–42pyr is 3325 Da, m/z for Aβ 1–42 is 4520 Da)

The TAT trojan sequence efficiently allowed the internalization of the proteins in primary cortical neurons. Confocal pictures showed that TAT-GFP was internalized within 1 h after the treatment (Additional file 1: Figure S1A, B). Optical sectioning showed GFP-rich endosome-like structures in the cytoplasm. TAT-GFP signal was also evident in live cells imaged 1 h after the treatment (Additional file 1: Figure S1C). GFP fluorescence was found both in the somas as well as in neurites. We also checked, with MTT assay, whether the mutant peptides were toxic in primary cortical neurons (Additional file 1: Figure S1D). After 24 h treatment with 2 μM concentration, no toxic effect was observed. Mature cortical neurons were then treated for 24 h with 1 μM constitutively active (CA) double mutants (Rac1-L61F37A or Rac1-L61Y40C), Rac1-WT, or Rac1-DN and stained for F-actin to verify that the peptides were active (Fig. 2a). Both CA mutants increased F-actin reactivity compared to controls, Rac1-WT, and Rac1 DN. Rac1-DN reduced F-actin levels as expected. Staining against 4G8 antibody showed that both CA mutants enhanced the immunoreactivity of Aβ and/or its precursor compared to controls, Rac1-WT, and Rac1-DN (Fig. 2a). The second mutation of the CA proteins, F37A or Y40C, did not exert any differential effect, indicating that the observed effect on Aβ metabolism is dependent on the Q61L mutation, which the double mutants had in common. To determine which Aβ isoform was increasingly generated after the Rac1 peptide treatments, we performed immunoproteomic analysis, using surface-enhanced laser desorption/ionization time of flight mass spectrometry (SELDI-TOF MS). Human SH-SY5Y neuroblastoma cells were treated with TAT-Rac1 mutant proteins or vehicle: the lysate analysis revealed the presence of two main Aβ peptides, Aβ_1–42_ and Aβ_11–42_ pyr, after Rac1-L61F37A treatment (Fig. 2b).

To evaluate the directionality of the signalling, we also checked whether Aβ administration was able to modulate Rac1. We used synthetic Aβ_1–42_, which we allowed to aggregate at 4 °C for 12 h. To test Aβ_1–42_ toxicity, an MTT assay was performed (Additional file 1: Figure S2A). Mature cortical neurons, cultured for 10 days in vitro (DIV), were incubated for 24 h with different Aβ_1–42_ concentrations, ranging from 0.1 μM to 1 μM. The preparation induced a significant toxicity at 0.1, 0.5, and 1 μM. A dot-blot assay verified the presence of a detectable oligomeric population, using two different antibodies: 6E10 and A11 (Additional file 1: Figure S2B). 6E10 is reactive to the amino acid residues 1–16 of Aβ. It is sequence-specific and recognizes both fibrillar and oligomeric forms as well as the precursor form. A11 antibody selectively recognizes amino acid sequence-independent oligomers but does not recognize monomers or mature fibers. All Aβ_1–42_ samples were 6E10 and A11 positive, confirming that the preparations contained oligomers (representative image of the dot-blot assay).

Primary cortical neurons were treated with 0.1 μM Aβ_1–42_ at different time points: 1 h, 3 h, 6 h or 24 h. After treatments, cells were fixed and immunostained against the active form of Rac1 protein (Rac1-GTP), neurofilaments, and F-actin. After 0.1 μM Aβ_1–42_ administration, no differences were observed in Rac1 activation or localization in all the time points analysed. From the images acquired, F-actin seemed also not affected by the treatment (Additional file 1: Figure S2C). A few clots were observed along the neurites in the neurofilament staining after Aβ_1–42_ administration, at both concentrations, confirming the toxicity measured with the MTT assay. These findings indicate that the interference with Rac1 signalling promotes an increased APP processing.

### Rac1 perturbation affects SET translocation and results in tau hyperphosphorylation

**Fig. 3:**
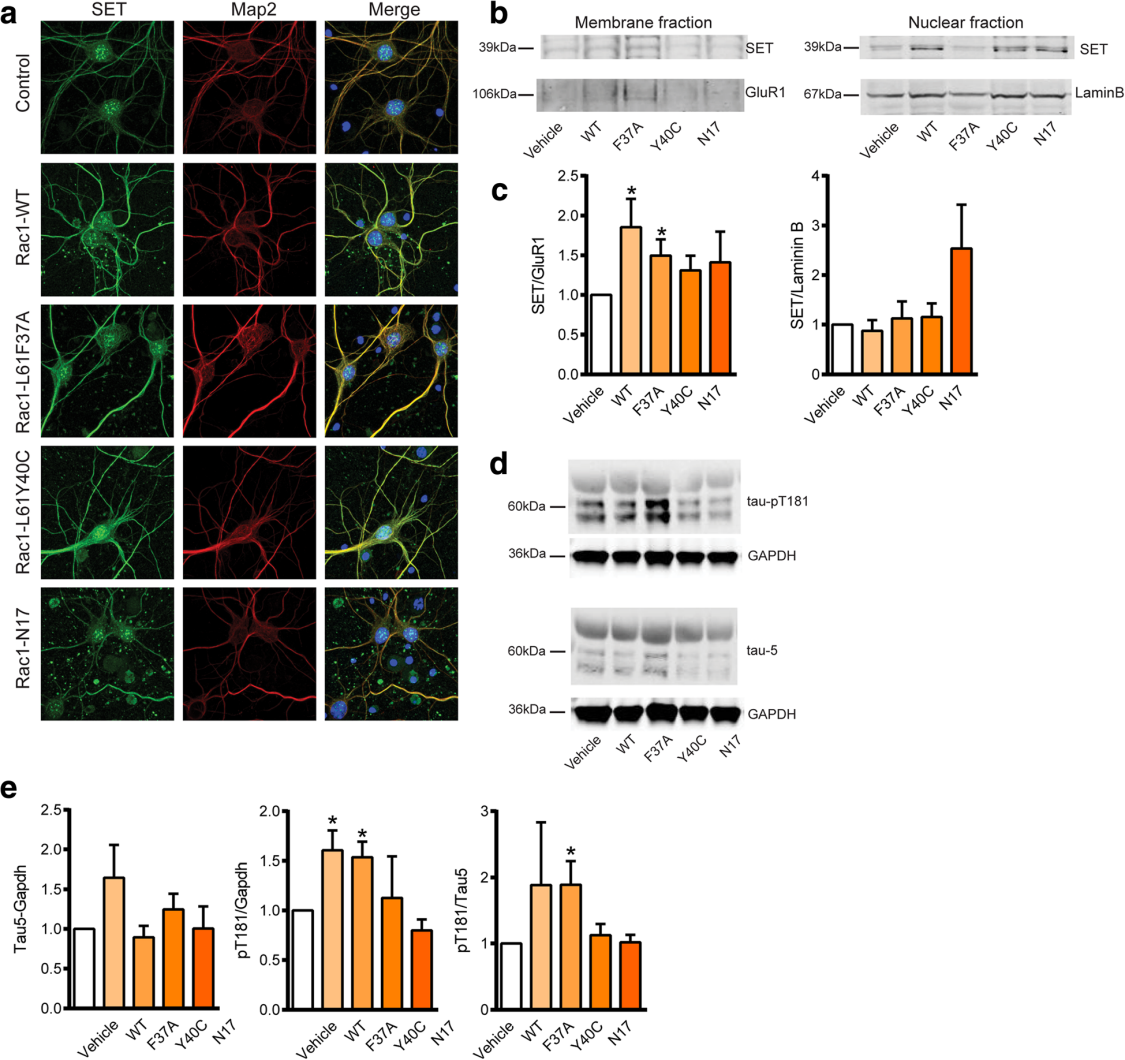
Rac1 mutant peptides induce tau hyperphosphorylation mediated by SET translocation. **a** Primary cortical neurons treated between DIV11 and DIV14 with Rac1 mutant peptides. After 24 h, cells were fixed and stained to visualize SET, dendrites (MAP2), and nuclei (DAPI). These representative images were obtained from one of three independent experiments. Scale bar 10 μm. **b-c** Representative blots and densitometry of subcellular fractionation indicating the levels of SET in the membrane (SET/GluR1) and nuclear fractions (SET/LaminB) in the same conditions as in A. 15 μg of protein lysate were loaded for the nuclear fraction. Due to the low yield, the membrane fraction was not quantified. **d-e** Representative blots and corresponding quantification of tau pT181 phosphorylation and Tau-5 normalized against GAPDH levels, and pT181/Tau5. ~ 7 μg of protein lysate were loaded

**Fig. 4:**
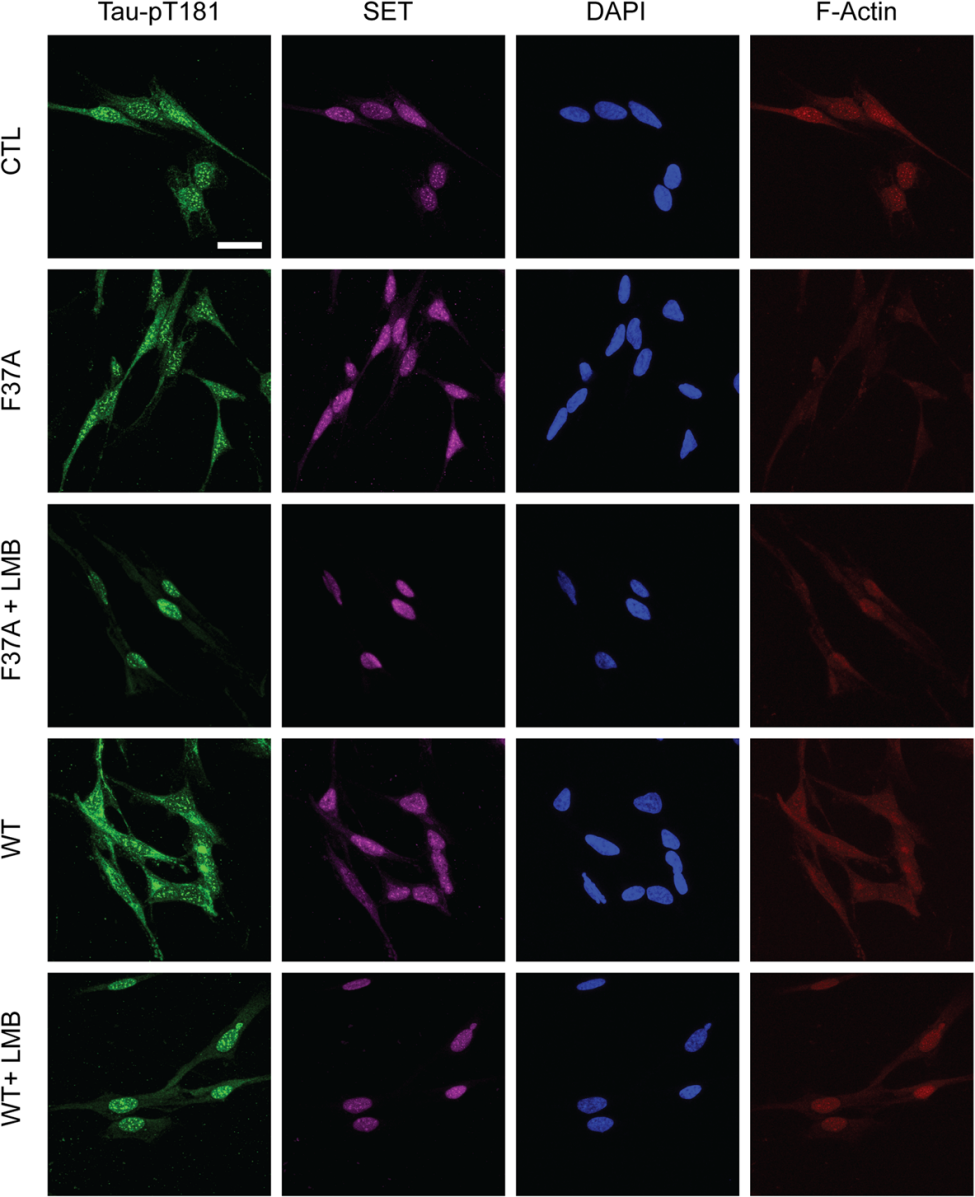
The nuclear transporter inhibitor LMB blocks Rac1-induced translocation of SET*.* SH-SY5Y cells at 10 days of RA differentiation were treated with Rac1-WT and Rac1-L61F37A, with or without LMB in order to block Rac1-peptide mediated SET translocation. After 48 h, cells were fixed and stained against pT181 tau epitope (green), SET (magenta), F-actin (red), and DAPI (blue) was used to visualize nuclei. Representative images show that, in control condition SET expression is restricted to the nucleus. After Rac1-WT and Rac1-L61F37A treatments, SET presence is observed also outside cell nuclei, whereas SET translocation doesn’t occur when LMB is added together with Rac1 mutant treatments. In the same way, tau phosphorylation at the epitope pT181 is decreased in presence of LMB. Scale bar 25 μm

Next, we evaluated whether Rac1 mutant peptides were able to interfere with tau phosphorylation. Neurons treated for 24 h with TAT-Rac1 mutants were stained against SET. SET, which is normally localized in the nucleus, translocated to the cytoplasm in AD brains. Its translocation from the nucleus to the cytoplasm was shown in AD temporal cortex and hippocampus, compared to age-matched controls [62]. Interestingly, the administration of both Rac1-WT and CA mutants was able to elicit the translocation of SET from the nucleus to the neurites (Fig. 3a). These results indicate that the protein itself is sufficient to induce SET translocation. To quantitative confirm this observation, we performed subcellular fractionation to purify the membranous and nuclear fractions. SET concentration in the membrane fraction significantly increased after Rac1-L61F37A and Rac1-WT treatments, while no change was observed in the nuclear fraction (Fig. 3b, c). Controls experiments to ensure the successful enrichment of the 2 fractions were performed in SH-SH5Y cells (Additional file 1: Figure S3). The abundancy of Lamin B was checked in the membrane fraction and the levels of GluR1 were assessed in the nuclear fraction. To check whether SET translocation resulted in an increased tau phosphorylation, cortical neurons were treated for 48 h with the peptides. We chose pT181 phospho-site as this is one of the major AD abnormally hyperphosphorylated sites regulated by PPA [66]. pT181/Tau5 was significantly increased after treatment with Rac1-L61F37A compared to vehicle treated cells (Fig. 3d, e). The use of a nuclear transporter inhibitor (LMB) reduced SET translocation from the nucleus to the membrane in SH-SY5Y when Rac1 peptides where administered. This impeded the increase in tau phosphorylation (Fig. 4).

As for Aβ, we checked whether tau-induced hyperphosphorylation altered Rac1 activation. We used okadaic acid (OA), a synthetic inhibitor of PP2A and PP1, which is a well-known tool to study AD pathology in vitro [46]. The evaluation of tau phosphorylation was performed for two of the main phosphorylated epitopes, pS262 and pS202 (Additional file 1: Figure S4A). Immunostaining after 6 h from OA treatment showed an enhanced tau immunoreactivity against both sites compared to the vehicle treated cells, with a pronounced accumulation in the somatodendritic compartment. The increased ratio pS262/tau was also detected via Western Blot at both time points (Additional file 1: Figure S4B, C). Neurons were also treated with 10 nM OA for 3 h or 6 h, and then analysed for Rac1-GTP by pull down assay. Rac1-GTP pull down assay showed no difference in the levels of activated proteins between OA treatment and control (Additional file 1: Figure S4D-E). In addition, the total expression of the protein was unchanged between the conditions. Overall, this data establishes a new direct pathway in which Rac1 induces SET translocation and, consequently, increases tau phosphorylation.

### Rac1 is biphasically altered in 3xTg-AD mice

**Fig. 5:**
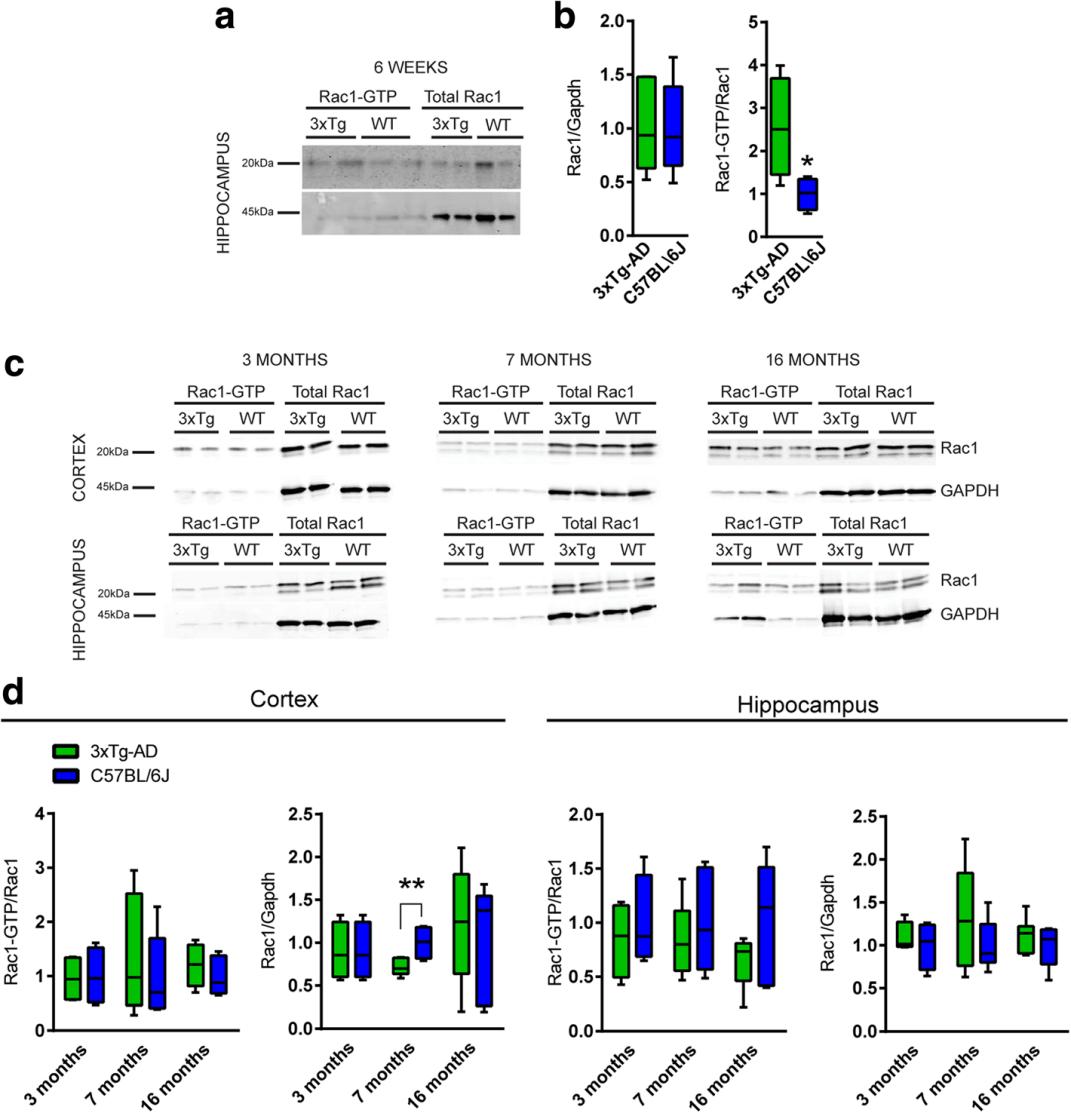
Rac1 is altered in 3xTg-AD mice. **a**, **c** Representative blots of active and total Rac1 protein levels in homogenates of 3xTg-AD mice and age-matched control mice. **b**, **d** Quantification of Rac1 protein activation (ratio between Rac1-GTP level and total Rac1), and total Rac1 level (total Rac1 on GAPDH ratio), at the 4 time points (6 weeks: C57BL/6 J *n* = 6, 3xTG-AD n = 6; 3 months: C57BL/6 J *n* = 4, 3xTG-AD n = 4; 7 months: C57BL/6 J *N* = 7, 3xTG-AD N = 7; 16 months: C57BL/6 J *n* = 5, 3xTG-AD n = 5). Box and whiskers graphs represent boxes as min to max values, whiskers as standard errors and means as black lines

We investigated whether the reduced Rac1 expression observed in post-mortem AD brains was also recapitulated in a mouse model of familial AD. The 3xTg-AD model was selected. Pull-down assay for Rac1 and Rac1-GTP was performed to evaluate Rac1 levels and activation in the cortex and hippocampus of control (C57BL/6 J) and 3xTg-AD mice. We first checked in young animals, at 6 weeks, and found increased ratio Rac1-GTP/Rac1 in the hippocampus of 3xTg-AD mice compared to age-matched controls (Fig. 5a, b). We next evaluated how the levels of the protein changed over time at 3, 7, and 16 months. The analysis revealed a statistically significant decrease in total Rac1 in the cortex of 3xTg-AD mice at 7-month-old compared to the controls. These findings suggest an abnormal activation of Rac1 at a very early stage of the pathology. This is followed by a decrease of the total level of the protein at a later stage, 7 months, when the cognitive impairment starts to become apparent according to published behavioral studies [60].

### Rac1-L61F37A mutant peptide rescues spine loss in 3xTg-AD mice

**Fig. 6:**
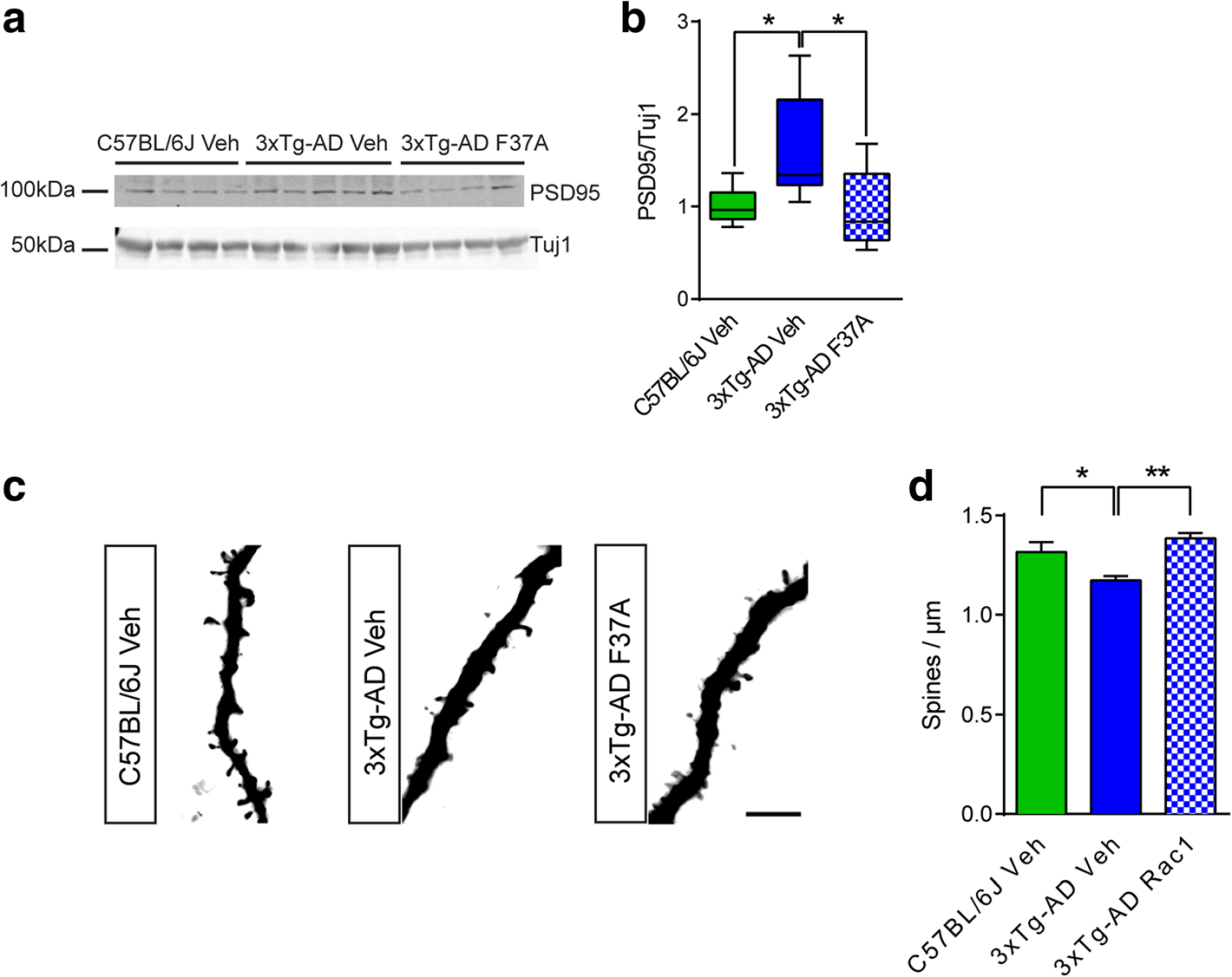
Rac1 administration rescued dendritic impairment. **a** Representative immunoblots and (**b**) densitometry of PSD95 were normalized on Tuj1 in cortical homogenates of control mice (C57BL/6 J treated with vehicle) and 3xTg-AD mice treated with vehicle or with Rac1-L61F37A mutant peptide (100 μM) at 7 months old. Box and whiskers graphs represent boxes as min to max values, whiskers as standard errors and means as black lines. 10 μg of protein lysate were loaded. **c** Representative Golgi-cox stained dendrite portions with spines and (**d**) quantification of spine density (numbers of spine per μm of dendrite length) in the three condition analyzed (C57BL/6 J treated with vehicle: 1.32 ± 0.05 spines/μm; 3xTg-AD treated with vehicle: 1.17 ± 0.02 spines/μm; and 3xTg-AD treated with vehicle: 1.39 ± 0.03 spines/μm). The data represented are mean ± SEM

Since Rac1 decreased in 7-month-old 3xTg-AD mice, we administered Rac1-L61F37A to evaluate its potential effect in ameliorating the known synaptic impairments [5]. We evaluated Rac1-L61F37A effect on the expression levels of PSD95 by Western Blot (Fig. 6a, b). Three experimental groups of animals were tested: C57BL/6 J mice treated with vehicle, 3xTg-AD mice treated with vehicle, and 3xTg-AD mice treated with Rac1-L61F37A (*n* = 7–9 animals per group). We observed a significant increase of the post-synaptic marker PSD95 in cortical homogenate of 7-month-old 3xTg-AD mice compared to controls. Importantly, after Rac1-L61F37A intranasal treatment, PSD95 levels normalized back to the control levels.

In order to analyze in more details the effect of Rac1-L61F37A mutant treatment, we evaluated the spine density in the 3 different groups. A total mean number of 5056 ± 1158.04 spines per animal were counted on mean dendrite length of 3903.49 ± 888.59 μm per animal, from 12 animals. Neuronal dendrites were acquired from four different cortical areas (primary motor cortex, premotor motor cortex, posterior parietal area and visual cortex) in each animal: C57BL/6 J treated with vehicle (*N* = 4), 3xTg-AD treated with vehicle (N = 4) and 3xTg-AD treated with Rac1-L61F37A (N = 4). 3xTg-AD mice showed a significant decrease in cortical spine density at 7-month-old respect to control mice (Fig. 6c-d). Rac1-L61F37A intranasal treatment in 3xTg-AD increased significantly spine density respect to age-match vehicle-treated mice, restoring spine density at the same level of control mice. This data indicates the beneficial effect of timely activating Rac1 signaling to reverse spine and synaptic abnormalities in a disease-relevant animal model.

### Pathways analysis indicates the interactome connecting Rac1 to tau and APP

**Fig. 7:**
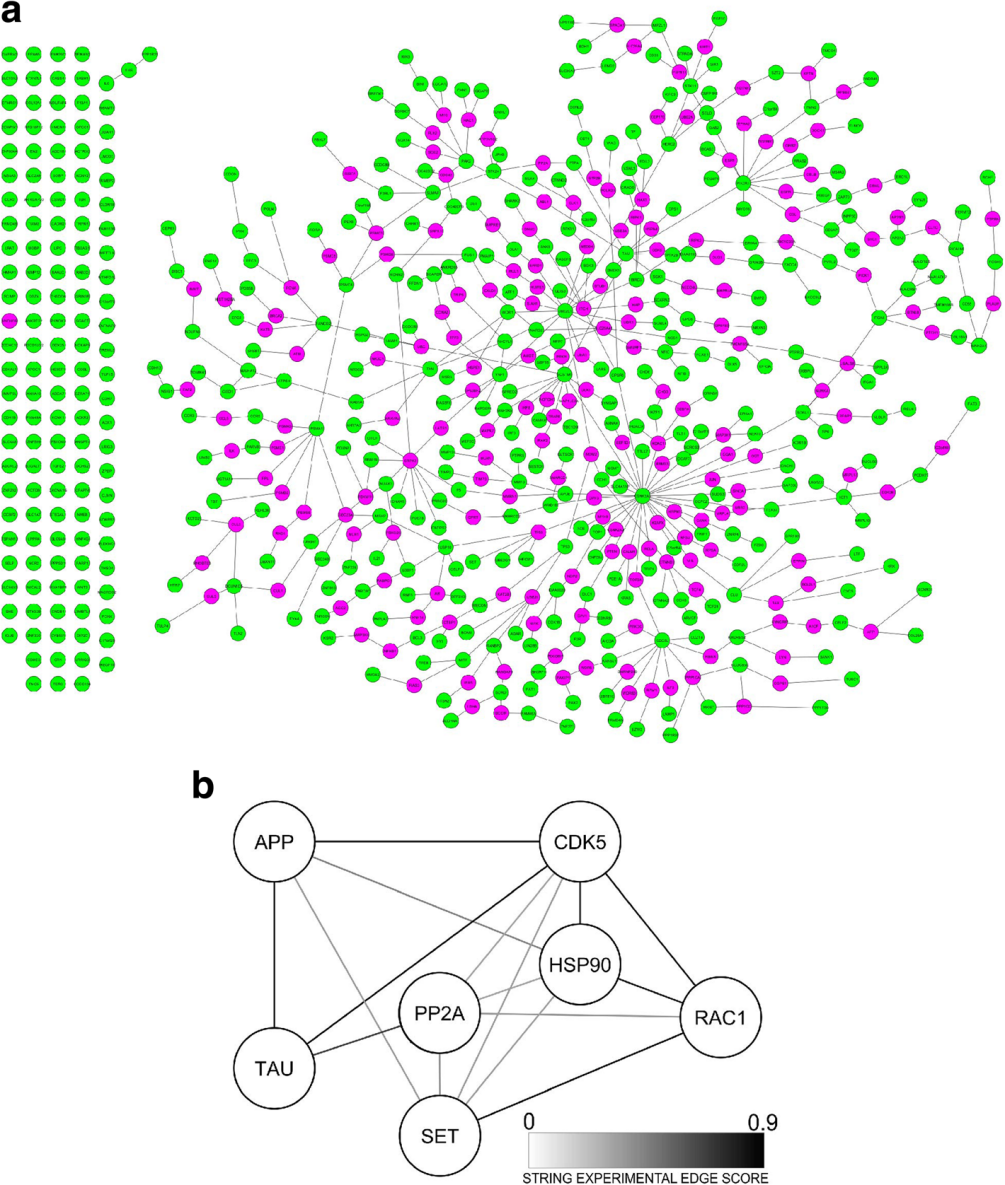
Rac1 is linked to PP2A in protein network analysis. **a** ANAT network starting from the AD GWAS genes plus SET and PP2A (in green) leading to the addition of high-confidence connecting genes (in purple). **b** Rac1, SET, PP2A shortest paths to either APP or Tau according to the STRING network based on GWAS AD plus ANAT high confidence interactors genes. Edges score opacity mapping STRING experimental score

To further characterize functional interactions between Rac1 and AD relevant proteins we performed ANAT analysis (Fig. 7a). In the resulting network, Rac1 is connected to the GWAS identified genes PAK2 [23], CHN2 [19], and IQGAP2 [76]. PAK2 is a well know Rac1 activator, CHN2 is an inhibitor upon EGF receptor stimulation in fibroblast-like cell lines cells [14], and IQGAP2 modulates Rac1 activity [13]. These proteins are all involved in cytoskeleton reorganization [21].

To deepen the analysis and to expand interactomic consistency to the network, the list of 720 GWAS identified genes, plus SET, PP2A and the 182 high confidence interactors were submitted to STRING. Of the 269 GWAS genes ANAT did not recognize, STRING recognized only 20, confirming the set of GWAS genes of interest. The interactions of Rac1 with PAK2, IQGAP2, and CHN2 were confirmed.

The neighborhood of Rac1, PP2A, and SET was selected from the STRING interactome. As recommended by STRING for higher confidence, we kept edges with a “STRING combined score” greater or equal to 0.7. The resulting network of 113 genes included also APP and TAU. The 113 set of genes were analyzed with ClueGO [7] for a Gene Ontology biological process (BP) evaluation. The first 10 BP classes sorted by number of interested genes resulting from the analysis contain between 81 and 31 genes and 4 of them are cytoskeleton-related: “adherens junction assembly” (47 genes), positive regulation of protein complex assembly (43 genes), dendritic spine development (34 genes) and stress fiber assembly (31 genes).

To increase the resolution of the analysis, we calculated the shortest paths connecting Rac1, SET, PP2A, and APP or tau. According to STRING results, these proteins are connected through CDK5 and HSP90AA1 (HSP90A), both added by ANAT to the genes set to build a possible high confidence interactome (Fig. 7b). CDK5 is related to several of the aforementioned proteins. HSP90A was recently identified as a promising AD treatment target [8] and a HSP90A knockout has been associated with Rac1 down regulation [20]. It also interferes with PP2A mediated AKT phosphorylation with implications for AD [35].

CDK5 silencing has a Rac1-mediated neuroprotective effect [54]. SET is required for the stimulatory effect on the CDK5 region p35(nck5a) and CDK5 phosphorylates APP and TAU [26, 29], and it has been suggested that PP2A might act in functional association with GSK3 in tau hyperphosphorylation [53].

## Discussion

Members of the Rho-GTPase family have been previously connected to pathogenic events contributing to synaptic deficits in AD. Our data describes a putative pathway in which Rac1 is up-stream and timely elicits the alterations of AD relevant proteins. The selective activation of Rac1 signalling enhanced Aβ levels and promoted SET translocation from the nucleus to the plasma membrane. The directionality of this alteration was confirmed as Aβ administration and tau-induced hyperphosphorylation did not perturb Rac1 cellular distribution or activation. At the same time, Rac1 increased in young 3xTg-AD mice and later decreased at 7 months. At this latter time point, the intranasal administration of Rac1 active peptide restored spine loss. This indicates that the involvement of Rac1 in AD pathological cascade of events is rather complex. The contribution of Rac1 to these apparently contradicting pathways might be explained with the tight spatiotemporal regulation of the Rho-GTPases. Previous studies showing contrasting data on the levels of Rac1 in AD versus age-matched controls might be reconsidered in this light. Rac1 was reported to be increased [49], or decreased [36] in AD autoptic brain samples. A closer look into the pathological diagnoses of the mentioned studies shows that Rac1 increased when patients with mild AD were selected [49], meanwhile it decreased in samples with NTF stage V or VI [36] because of the extensive neuronal death. In the present study, we found a reduction of Rac1 protein levels in human AD brain. This decrease was accompanied by an increased protein plasma levels in AD patients with the most severe cognitive decline (MMSE < 18). In addition, Rac1 plasma levels weakly correlated with the cognitive decline in AD, thus suggesting that this protein might represent a marker of AD disease progression: further investigation are mandatories to confirm these preliminary results. At this stage, therapeutic intervention boosting Rac1 signalling to support spine maintenance might represent an interesting option. 3xTg-AD mice treated for 2 weeks at 6.5 months with Rac1-L61F37A showed a rescue of spine deficits. Both male and female 3xTg-AD mice showed a subtle deficit in spatial learning and memory exactly at 6.5 months of age [60], this underlying the spine impairment. Rac1-L61F37A peptide was previously shown to boost cell survival and regeneration after optic nerve crush by the activation of the Pak\MEK\Erk pathway [30]. The protective effect might also be ascribed to the release of neurotrophic factors as activation of Erk1/2 resulted in the secretion of endogenous CNTF [40]. Importantly, intranasal treatment with Rac1-L61F37A did not significantly interfere with tau phosphorylation and APP processing when administered in 3xTg-AD (data not shown). Rac1- L61F37A also normalized the levels of PSD95 proteins in 3xTg-AD compared to 3xTg-AD treated with vehicle. It was previously reported that PSD95 decreased in 3xTg-AD 7 month-old animals [55]. One of the reasons for this discrepancy might lie in the different loading controls used (Tuj1 in this study versus actin in Revilla et al.). Since we administered an actin modulating protein, Tuj1 seemed a better choice. Moreover, many papers have described how AD impairs actin stability [48] and its levels might change over the course of the pathology.

The pathway analysis offered a high-level view of the pathways connecting Rac1 to AD relevant proteins and highlighted the strong interaction between Rac1 and tau through SET and PP2A. The use of mutant peptides allowed us to better dissect Rac1 signaling, which is executed by several effectors. In these mutants, the L61 mutation, which tonically activated the protein, was coupled to a second mutation (F37A or Y40C) that gave signal specificity [30]. The Y40C blocked the binding to PAK and JNK mediated pathways meanwhile, F37A activated them. The specific effect of Rac1-L61F37A on tau hyperphosphorylation might be mediated by the effector protein PAK. Rac1-induced PAK activation has been shown to activate p38MAPK [75], which phosphorylates tau [72].

The reduction of PP2A activity via SET has been shown to affect APP regulation [24]. Coherently, we observed that Rac1-L61F37A was also the most effective mutant in determining an increase of Aβ fragments 11–42pyr and 1–42. Overexpression of both C and N terminals of SET in rats determined Aβ accumulation starting from 4-month old rats [9]. When we tested whether Rac1 could be altered following Aβ administration, we could not observe any impairment. Other studies used synthetic Aβ peptide and showed a consequent Rac1 activation. However, in these studies, the used Aβ concentration was in the μM range (above 1 μM) [34, 39]. Concentrations higher than 1 μM have been defined by many as “supraphysiological” [22] and the data obtained thus require careful consideration.

Despite the use of Rac1 mutant peptides, which allowed different signaling cascades to be triggered, they do not provide insights into the kinetic of the activation. As already proposed [50], Rho-GTPases alteration needs to be studied with tools allowing to follow their spatiotemporal dynamics. The different functions of Rac1 suggest a highly controlled regulation, which is also dependent on the cellular compartment. In this regard, new imaging tools based on sophisticated fluorescent biosensors can help to resolve the dynamics of these proteins, which are activated on a micrometer length and sub-minute time scales [41, 71, 73]. These tools might highlight even more subtle defects in either their compartmentalization or crosstalk between the family members.

Additional studies are clearly necessary to further unravel this intricate signaling. Elucidating the molecular mechanisms underlying the loss of spines is certainly essential for the development of disease-modifying therapeutics. Moreover, the possibility of further investigating Rho-GTPase members as potential indicator of disease progression for AD in plasma represents an interesting option. We showed here that Rac1 increased in AD patients with MMSE< 18 and, in a recent work, that Cdc42 decreased in fronto-temporal dementia patients [56].

## Conclusion

Rac1 might have a role in AD as a triggering co-factor, participating both to Aβ and tau alteration. However, at a later stage of the pathology, it might represent a potential therapeutic target due to its beneficial effect on dendritic spine dynamics.

## Additional file

Additional file 1: **Figure S1.** Rac1 mutant peptides have high penetration due to the TAT sequence. (**A-C**) Representative confocal images of cortical neurons treated at DIV3 with different concentrations of TAT-GFP: 5 μM (**A**), 10 μM (**B, C**). After treatment, cells were fixed and stained for visualization of dendrites (MAP2) and nuclei (DAPI). Confocal analysis showed that TAT-GFP was internalized (single plane), also in live cells directly imaged 1h after treatment. Scale bars 10 μm. (**D**) MTT assay on primary cortical neurons after 24h from the administration of 2 μM Rac1 mutant peptides. The cell viability is expressed as % as compared to control. The data represented are mean ±SEM of four independent experiments, each done in triplicate. **Figure S2.** Aβ_1-42_ administration does not interfere with Rac1 localization or activation. (**A**) MTT assay on primary cortical neurons after 24h Aβ_1-42_ treatment at the indicated concentrations The Aβ peptide suspension was incubated 12h at 4°C prior treatment. The cell viability is expressed as % as compared to control. The data represented are mean ±SEM of four independent experiments, each done in triplicate. One-sample *t* test to a hypothetical mean of 100% was performed. (**B**) Representative dot-blot analysis of Aβ_1-42_ preparations with 6E10 and A11 antibodies. The protein concentration was 0.12 μg for 6E10 and 0.72 μg for A11 (**C**) Representative confocal images of primary cortical neurons treated with 0.1 μM Aβ_1-42_ between DIV11 and DIV14. Cells were stained against Rac1-GTP, F-actin, and neurofilament. Scale bars 30 μm. **Figure S3.** Efficacy of the subcellular fractionation. Representative blots of the subcellular fractionation experiments showing the levels of GluR1, LaminB, and SET in the membrane and nuclear fractions of SH-SY5Y cells. Four independent samples were assessed for the 2 fractions. **Figure S4.** Tau induced hyperphosphorylation does not alter Rac1 levels or activation. (**A**) Representative confocal pictures of mature cortical neurons treated with 10nM OA for 6h and immunostained against pS262 tau. Scale bar 30 μm. (**B-C**) Tau pS262 phosphorylation was analysed by western blot after 3 and 6h from OA administration. The data represented are mean with SEM of four or six independent experiments (3h treatment n=6, 6h treatment n=4). (**D-E**) Rac1-GTP pull done assay was performed after 3 and 6h from OA administration. The data represented are mean with SEM of three independent experiments. ns, not significant. Asterisks indicate unspecific bands. (DOCX 3215 kb)
