# Supplementary material for: Rac1 activation links tau hyperphosphorylation and Aβ dysmetabolism in Alzheimer’s disease
Source: Acta Neuropathol Commun. 2018 Jul 13;6:61. doi: 10.1186/s40478-018-0567-4 (PMC6045891; doi:10.1186/s40478-018-0567-4)
Supplement: Supplementary file 1 — Figure S1. Rac1 mutant peptides have high penetration due to the TAT sequence. (A-C) Representative confocal images of cortical neurons treated at DIV3 with different concentrations of TAT-GFP: 5 μM (A), 10 μM (B, C). After treatment, cells were fixed and stained for visualization of dendrites (MAP2) and nuclei (DAPI). Confocal analysis showed that TAT-GFP was internalized (single plane), also in live cells directly imaged 1h after treatment. Scale bars 10 μm. (D) MTT assay on primary cortical neurons after 24h from the administration of 2 μM Rac1 mutant peptides. The cell viability is expressed as % as compared to control. The data represented are mean ±SEM of four independent experiments, each done in triplicate. Figure S2. Aβ1-42 administration does not interfere with Rac1 localization or activation. (A) MTT assay on primary cortical neurons after 24h Aβ1-42 treatment at the indicated concentrations The Aβ peptide suspension was incubated 12h at 4°C prior treatment. The cell viability is expressed as % as compared to control. The data represented are mean ±SEM of four independent experiments, each done in triplicate. One-sample t test to a hypothetical mean of 100% was performed. (B) Representative dot-blot analysis of Aβ1-42 preparations with 6E10 and A11 antibodies. The protein concentration was 0.12 μg for 6E10 and 0.72 μg for A11 (C) Representative confocal images of primary cortical neurons treated with 0.1 μM Aβ1-42 between DIV11 and DIV14. Cells were stained against Rac1-GTP, F-actin, and neurofilament. Scale bars 30 μm. Figure S3. Efficacy of the subcellular fractionation. Representative blots of the subcellular fractionation experiments showing the levels of GluR1, LaminB, and SET in the membrane and nuclear fractions of SH-SY5Y cells. Four independent samples were assessed for the 2 fractions. Figure S4. Tau induced hyperphosphorylation does not alter Rac1 levels or activation. (A) Representative confocal pictures of mature cortical neurons treated with [file 40478_2018_567_MOESM1_ESM.docx]

Rac1 activation links tau hyperphosphorylation and Aβ dismetabolism in Alzheimer’s Disease

**Supplementary Information**

**Supplementary Figure Legends**

**Fig. S1 Rac1 mutant peptides have high penetration due to the TAT sequence**

(**A-C**) Representative confocal images of cortical neurons treated at DIV3 with different concentrations of TAT-GFP: 5 µM (**A**), 10 µM (**B, C**). After treatment, cells were fixed and stained for visualization of dendrites (MAP2) and nuclei (DAPI). Confocal analysis showed that TAT-GFP was internalized (single plane), also in live cells directly imaged 1h after treatment. Scale bars 10 µm. (**D**) MTT assay on primary cortical neurons after 24h from the administration of 2 µM Rac1 mutant peptides. The cell viability is expressed as % as compared to control. The data represented are mean ±SEM of four independent experiments, each done in triplicate.

**Fig. S2 Aβ_1-42_ administration does not interfere with Rac1 localization or activation**

(**A**) MTT assay on primary cortical neurons after 24h Aβ_1-42_ treatment at the indicated concentrations The Aβ peptide suspension was incubated 12h at 4°C prior treatment. The cell viability is expressed as % as compared to control. The data represented are mean ±SEM of four independent experiments, each done in triplicate. One-sample *t* test to a hypothetical mean of 100% was performed. (**B**) Representative dot-blot analysis of Aβ_1-42_ preparations with 6E10 and A11 antibodies. The protein concentration was 0.12 µg for 6E10 and 0.72 µg for A11 (**C**) Representative confocal images of primary cortical neurons treated with 0.1 µM Aβ_1-42_ between DIV11 and DIV14. Cells were stained against Rac1-GTP, F-actin, and neurofilament. Scale bars 30 µm.

**Fig. S3 Efficacy of the subcellular fractionation**

Representative blots of the subcellular fractionation experiments showing the levels of GluR1, LaminB, and SET in the membrane and nuclear fractions of SH-SY5Y cells. Four independent samples were assessed for the 2 fractions.

**Fig. S4 Tau induced hyperphosphorylation does not alter Rac1 levels or activation**

(**A**) Representative confocal pictures of mature cortical neurons treated with 10nM OA for 6h and immunostained against pS262 tau. Scale bar 30 µm. (**B-C**) Tau pS262 phosphorylation was analysed by western blot after 3 and 6h from OA administration. The data represented are mean with SEM of four or six independent experiments (3h treatment n=6, 6h treatment n=4). (**D-E**) Rac1-GTP pull done assay was performed after 3 and 6h from OA administration. The data represented are mean with SEM of three independent experiments. ns, not significant. Asterisks indicate unspecific bands.

**Supplementary Figures**

**Figure S1**

**
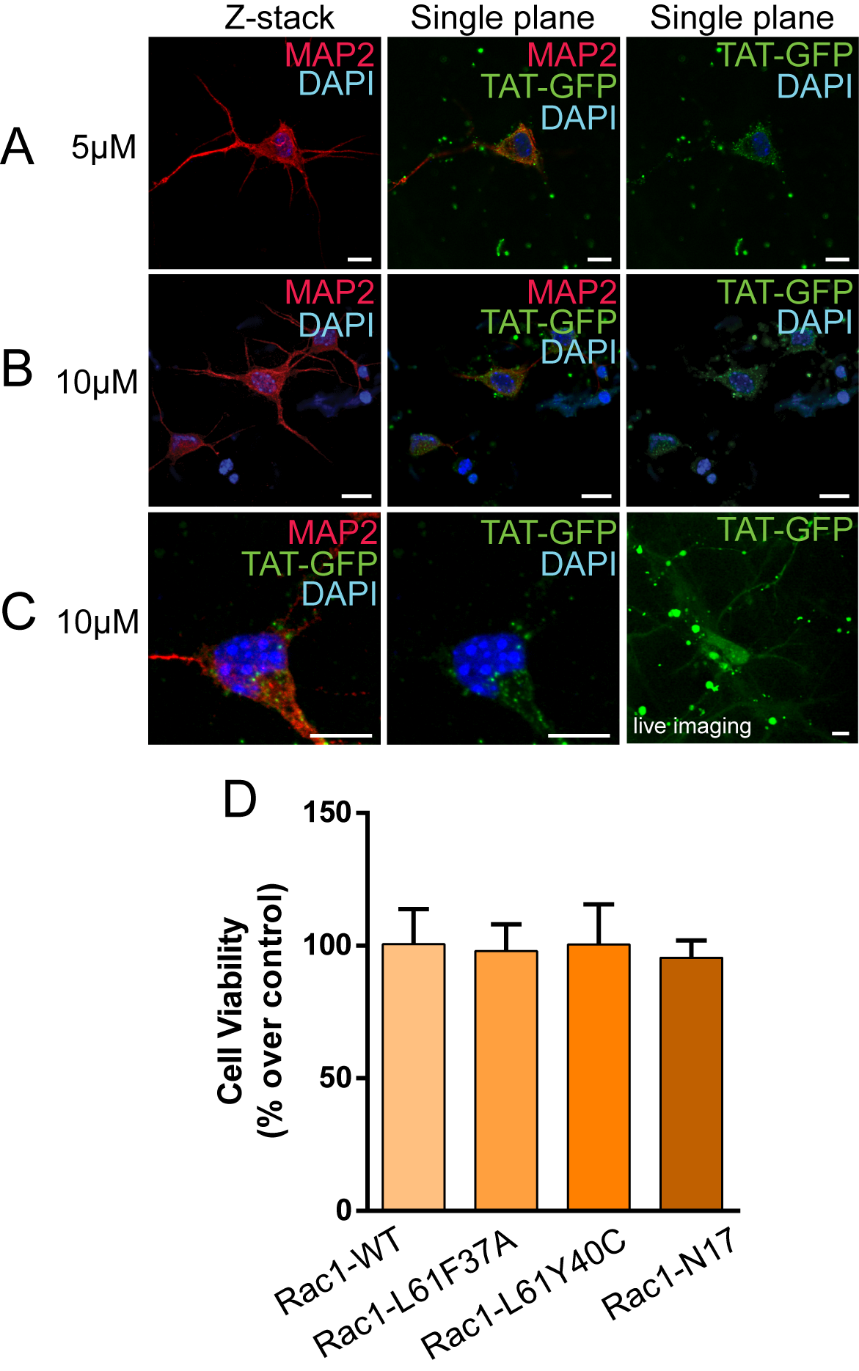
**

**Figure S2**


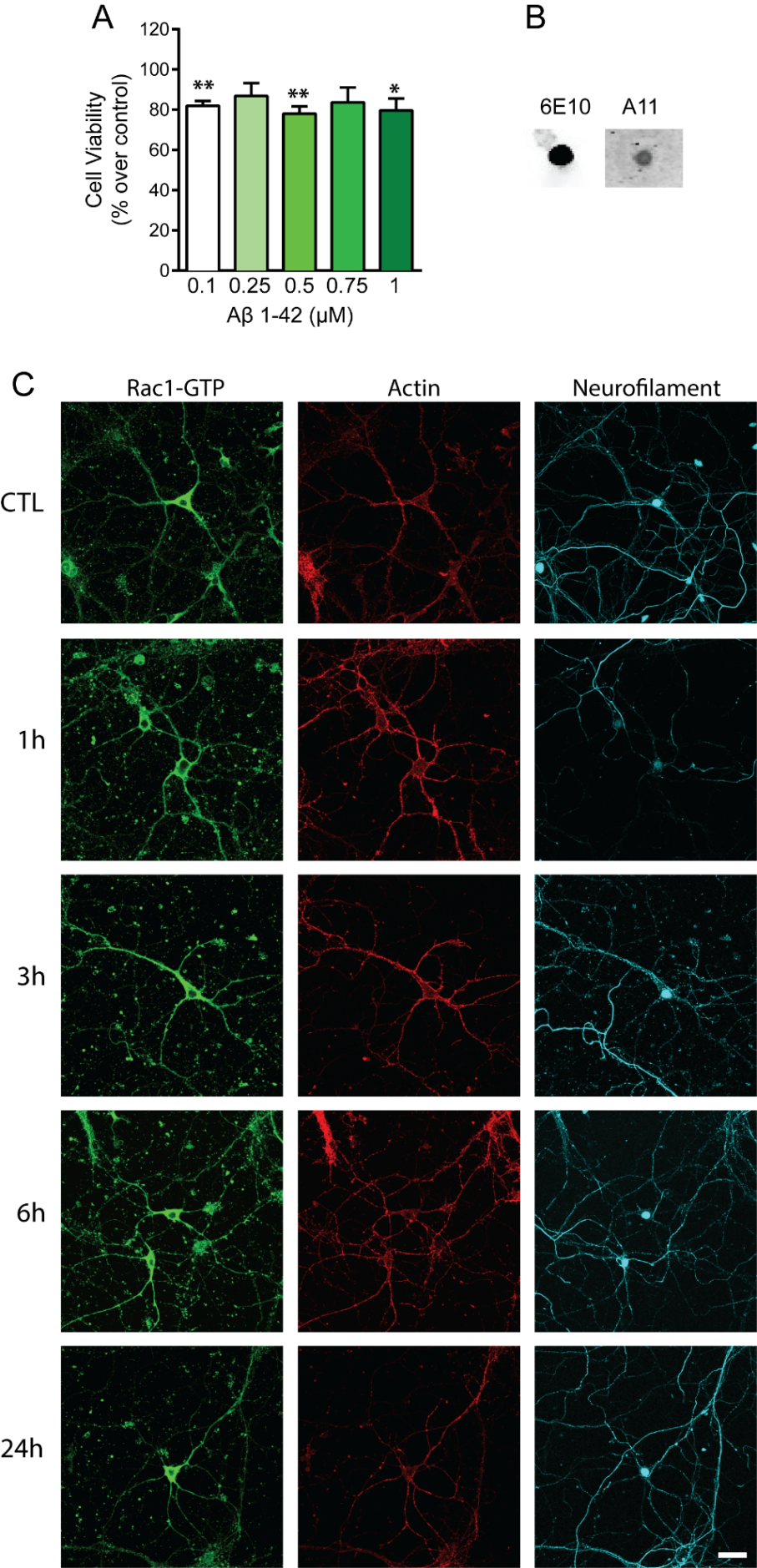


**Figure S3**

**
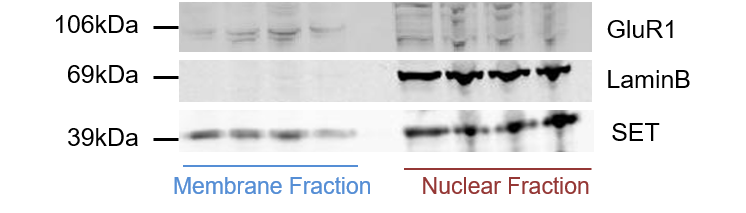
**

**Figure S4**

**
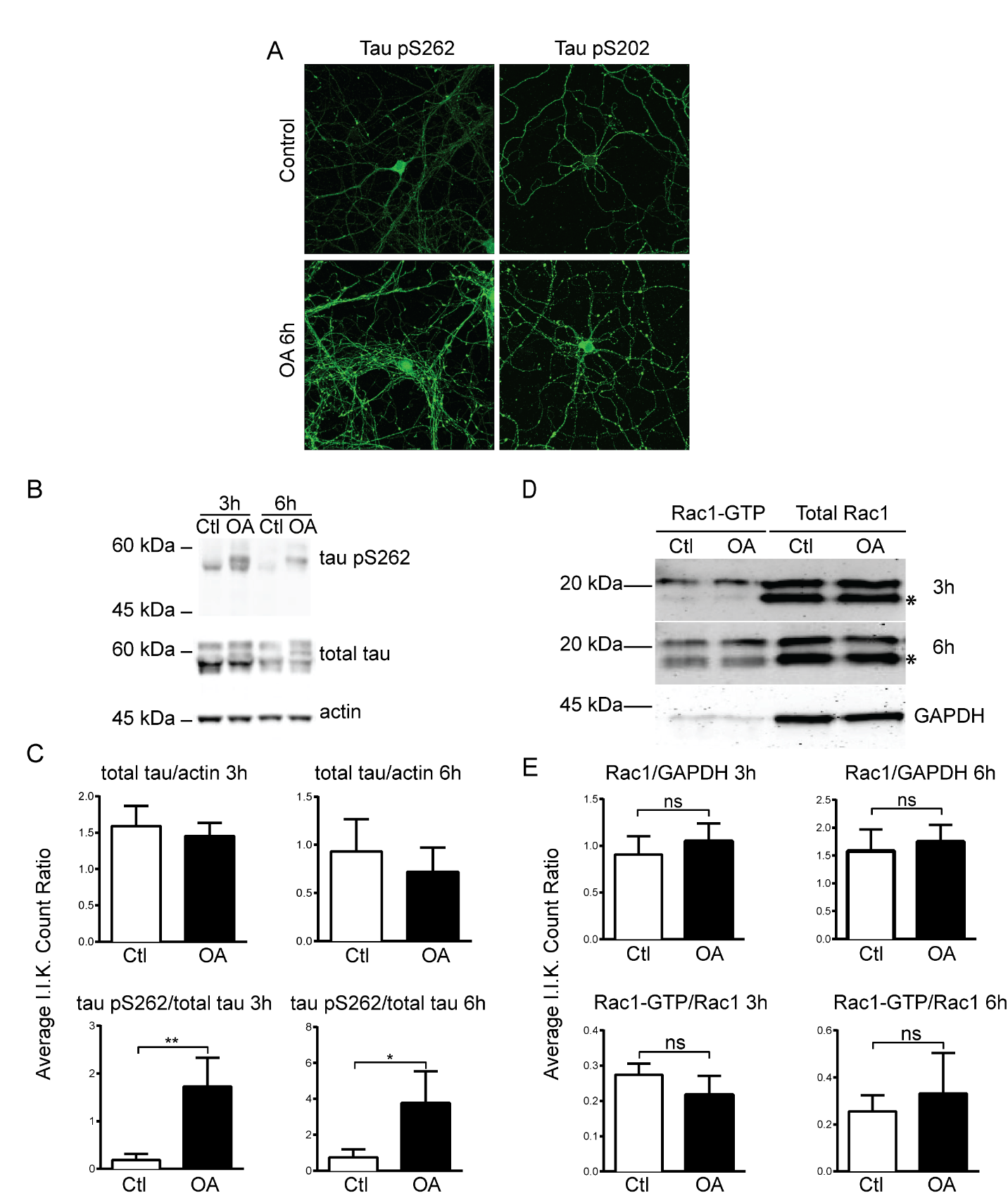
**
